# Localization of the Houdinisome (Ejection Proteins) inside the Bacteriophage P22 Virion by Bubblegram Imaging

**DOI:** 10.1128/mBio.01152-16

**Published:** 2016-08-09

**Authors:** Weimin Wu, Justin C. Leavitt, Naiqian Cheng, Eddie B. Gilcrease, Tina Motwani, Carolyn M. Teschke, Sherwood R. Casjens, Alasdair C. Steven

**Affiliations:** a Laboratory of Structural Biology Research, National Institute of Arthritis and Musculoskeletal and Skin Diseases, National Institutes of Health, Bethesda, Maryland, USA; b Division of Microbiology and Immunology, Department of Pathology, University of Utah School of Medicine, Salt Lake City, Utah, USA; c Department of Molecular and Cell Biology, University of Connecticut, Storrs, Connecticut, USA; d Department of Chemistry, University of Connecticut, Storrs, Connecticut, USA

## Abstract

The P22 capsid is a T=7 icosahedrally symmetric protein shell with a portal protein dodecamer at one 5-fold vertex. Extending outwards from that vertex is a short tail, and putatively extending inwards is a 15-nm-long α-helical barrel formed by the C-terminal domains of portal protein subunits. In addition to the densely packed genome, the capsid contains three “ejection proteins” (E-proteins [gp7, gp16, and gp20]) destined to exit from the tightly sealed capsid during the process of DNA delivery into target cells. We estimated their copy numbers by quantitative SDS-PAGE as approximately 12 molecules per virion of gp16 and gp7 and 30 copies of gp20. To localize them, we used bubblegram imaging, an adaptation of cryo-electron microscopy in which gaseous bubbles induced in proteins by prolonged irradiation are used to map the proteins’ locations. We applied this technique to wild-type P22, a triple mutant lacking all three E-proteins, and three mutants each lacking one E-protein. We conclude that all three E-proteins are loosely clustered around the portal axis, in the region displaced radially inwards from the portal crown. The bubblegram data imply that approximately half of the α-helical barrel seen in the portal crystal structure is disordered in the mature virion, and parts of the disordered region present binding sites for E-proteins. Thus positioned, the E-proteins are strategically placed to pass down the shortened barrel and through the portal ring and the tail, as they exit from the capsid during an infection.

## IMPORTANCE

While it has long been appreciated that capsids serve as delivery vehicles for viral genomes, there is now growing awareness that viruses also deliver proteins into their host cells. P22 has three such proteins (ejection proteins [E-proteins]), whose initial locations in the virion have remained unknown despite their copious amounts (total of 2.5 MDa). This study succeeded in localizing them by the novel technique of bubblegram imaging. The P22 E-proteins are seen to be distributed around the orifice of the portal barrel. Interestingly, this barrel, 15 nm long in a crystal structure, is only about half as long *in situ*: the remaining, disordered, portion appears to present binding sites for E-proteins. These observations document a spectacular example of a regulatory order-disorder transition in a supramolecular system and demonstrate the potential of bubblegram imaging to map the components of other viruses as well as cellular complexes.

## INTRODUCTION

Double-stranded DNA viruses such as the tailed bacteriophages and herpesviruses encapsidate their genomes to a concentration approaching the maximum physically possible: ~400 mg/ml. The intravirion DNA, as visualized by cryo-electron microscopy (cryo-EM), is closely packed in concentric, coaxially coiled shells (e.g., see references 1 and 2). Although already in 1952 Hershey and Chase (3) demonstrated that phage T2 virion DNA and not “the bulk of the sulfur-containing protein” is injected into target cells to identify DNA as the genetic material, we now know that phage capsids also contain internal proteins that are expelled from the virion into the cell during infection and have functions that are needed for the infection to be productive (4–7). The capsid interior is shielded from the environment to protect the genome from marauding nucleases and other threats. The most likely exit pathway for such internal proteins is the portal channel through which DNA enters and exits the capsid. However, the diameters of globular proteins may exceed the internal diameter of the portal channel (~3.5 nm). The exit of these internal proteins from the capsid represents a remarkable case of molecular escape artistry that presumably depends on, among other factors, the locations of the ejection proteins at the time DNA release initiates. (Harry Houdini, the doyen of escape artists, had a vaudeville act in which he would extricate himself from seemingly impossible states of confinement—hence, for elusive proteins, the term “Houdinisome.”)

Unlike capsid proteins, whose precise ordering in icosahedral lattices has facilitated structural analyses to high resolution by cryo-EM, internal proteins have mostly been undetected in such studies because they are less well ordered and also because they are camouflaged by contrast-matching with the DNA (e.g., see references 8 and 9). An exception to this trend is phage T7, which has an internal “core” consisting of stacked rings of three proteins (10, 11). In the present study, we have investigated the internal proteins of P22, a short-tailed bacteriophage of *Salmonella enterica*.

Twelve different P22-encoded proteins are required for virion assembly, of which nine are present in the final particle (summarized in reference 12). Cryo-EM reconstructions of the capsid and virion (8, 13–16) and crystal structures of several virion proteins (17–19) have given a detailed account of the overall structure of the virion (8, 20). However, three components remain unaccounted for: gp7 (21 kDa), gp16 (64 kDa), and gp20 (50 kDa). These proteins are not required for virion assembly but are absolutely necessary for delivery of the viral DNA into host cells (21–24). Because they are ejected from the virion prior to or with the DNA during initiation of infection, they have been called ejection proteins, or E-proteins (25, 26).

The P22 capsid has T=7 architecture, and its capsid protein conforms to the canonical HK97 fold (27–29), augmented by an insertion domain (30–32). One vertex is occupied by the dodecameric portal ring, through which DNA is translocated into the capsid during assembly and through which it exits during infection. P22 has a relatively large portal protein (gp1 [82.7 kDa]) whose crystal structure (18) revealed a feature unique among known portal structures: its 123-amino-acid-long C-terminal region was visualized as an α-helical coiled coil in the form of a barrel 15 nm long with an internal diameter of ~3.5 nm. Sequence analysis (8) and cryo-EM reconstructions of the P22-like phages Sf6 (33) and CUS-3 (34) suggest that the barrel may be a common feature in this phage group. However, the disposition of these barrel domains within the mature P22 virion has not been established and is one of the questions addressed here.

As noted above, direct visualization of internal proteins by cryo-EM can be obscured by the surrounding DNA. Localization by such techniques as antibody labeling or trimming with proteases is not applicable because the agents responsible cannot penetrate the capsid to access the internal proteins. In these circumstances, “bubblegram imaging” (35–38) offers a possible approach. This technique exploits the products of radiation damage. The hydrogen gas released from ice-embedded proteins that are subjected to electron irradiation is eventually manifested in the generation of bubbles which are readily visible in micrographs (35, 39–41). Over the electron dose regimen used, 50 to 250 electrons/Å^2^, DNA appears to be relatively unaffected, an inference supported by X-ray diffraction studies (see reference 42), but—pertinent to the present application—the bubbling of proteins is accelerated by being embedded in DNA (36). Here we have applied this approach to phage P22. To aid in interpreting the images, we also analyzed three single mutants, each lacking one E-protein, and a triple mutant lacking all three, by bubblegram imaging and by SDS-PAGE.

## RESULTS

### Quantitation of E-proteins in wild-type and mutant P22 virions.

All previous genetic studies of the E-proteins were performed with nonsense or temperature-sensitive (*ts*) mutations. To avoid potential complications in the present experiments that might arise from encapsidation of “amber” fragments of gp20 (see Adhikari and Berget [43]) or gp7 (E. B. Gilcrease and S. R. Casjens, unpublished results), we constructed phages with deletions of each E-protein gene. These lethal mutations were engineered into P22 prophages, which were propagated as bacterial lysogens, and mutant virions were made upon inducing the prophages to lytic growth (see Text S1 in the supplemental material). Five kinds of virions (the wild type, a triple mutant lacking all three E-proteins, called TriΔ, and the three single-deletion mutants Δ7, Δ16, and Δ20) were purified according to standard procedures (see Text S1). As morphologically normal particles were produced in each case (see below), it follows that the E-proteins are not essential for morphogenesis but are incorporated, if available.

**FIG 1:**
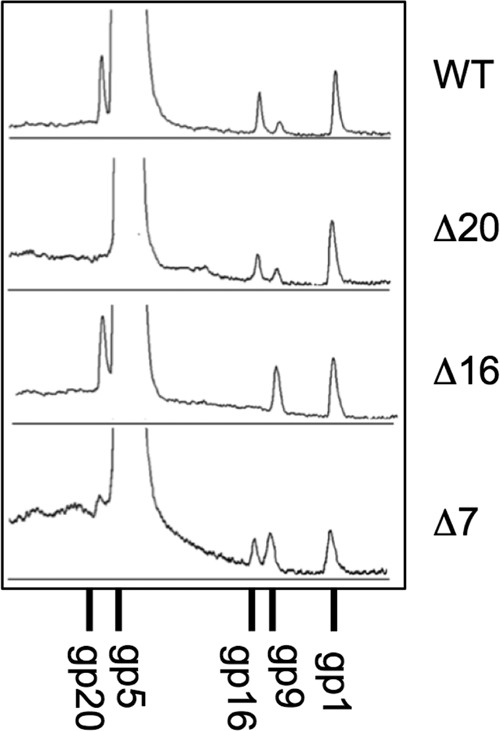
Quantitation of E-proteins in P22 virions. A 10% acrylamide SDS-PAGE gel of purified virions of the indicated genotypes was stained with Coomassie brilliant blue. Protein migration is right to left. The gel was imaged with a Bio-Rad Gel Doc apparatus, the image was converted to traces, and the gp1, gp16, and gp20 peaks were quantified with ImageJ. The scans were integrated across the whole lanes. (Integrations of narrower slices down the centers of the lanes gave indistinguishable results [data not shown].) The smaller proteins, gp7 and gp4, were quantified in parallel from 12.5% acrylamide gels of the same samples (not shown). The data were normalized according to molecular mass and 12 copies each of gp1 (for gp16 and gp20) and gp4 (for gp7). The resulting copy numbers are given in Table 1.

Hitherto, the E-protein copy numbers per virion have been estimated only roughly as about 20 (gp7), 6 (gp16), and 15 (gp20) (44). We used quantitative SDS-PAGE to revisit this question. Dye binding by each E-protein gel band was measured, and these data were converted to copy numbers, based on the assumption that the proteins have the same dye-binding affinity per molecular mass, and calibrated relative to the precisely known quotas of 12 copies each for the portal protein gp1 and the tail protein gp4 (8, 18). Typical gel traces are shown in Fig. 1, and the quantified results are given in Table 1. These data led to the following conclusions: to within experimental error, gp16 and gp7 are both present in the same copy number as the portal (12 subunits), and gp20 is somewhat more abundant at about 30 subunits. Thus, there are substantial amounts of E-proteins present in a wild-type virion: on average, 1.50 MDa of gp20, 0.77 MDa of gp16, and 0.25 MDa of gp7. Data from the single-deletion mutants indicate that the removal of one E-protein gene can affect the amounts of the other E-proteins. Specifically, deletion of gene 7 approximately halves the amount of gp16 and greatly reduces the amount of gp20 in virions, deletion of gene 16 results in moderately less gp20 but does not affect gp7, and deletion of gene 20 results in moderately less gp16 but does not affect gp7. Thus, there may be interactions between E-proteins during virion assembly.

**TABLE 1:** Copy numbers of E-protein subunits per P22 virion

Phage^a^	Copy no. of:			Total mass, MDa (fraction of WT)^c^
	gp20	gp16	gp7^b^	
WT	31.6 ± 3.0	11.9 ± 2.4	11.3 ± 2.7	2.52 (1.00)
Δ20	0	6.0 ± 0.8	9.9 ± 0.5	0.60 (0.24)
Δ16	22.4 ± 2.2	0	9.0 ± 1.3	1.29 (0.51)
Δ7	4.7 ± 1.3	5.6 ± 2.0	0	0.64 (0.25)

a Phages are all isogenic derivatives of P22 UC-739 (see text, Materials and Methods, and the supplemental material).

b A host protease removes the N-terminal 20 amino acids (aa) of the 229-amino-acid gp7 subunit before it assembles into virions (60). gp20 has 472 aa, and gp16 has 609 aa.

c The total measured mass of E-proteins per particle (fraction of wild-type in parentheses) is shown. SDS-PAGE gels of purified virions were stained with Coomassie blue and scanned (Fig. 1), and these data were converted to copy numbers as described in the legend to Fig. 1. The numbers listed are (max + min)/2 ± (max − min/2), where max and min are the highest and lowest values in a given set of measurements. Each set included three or more measurements.

### Mutant virions vary in their bubbling thresholds.

**FIG 2:**
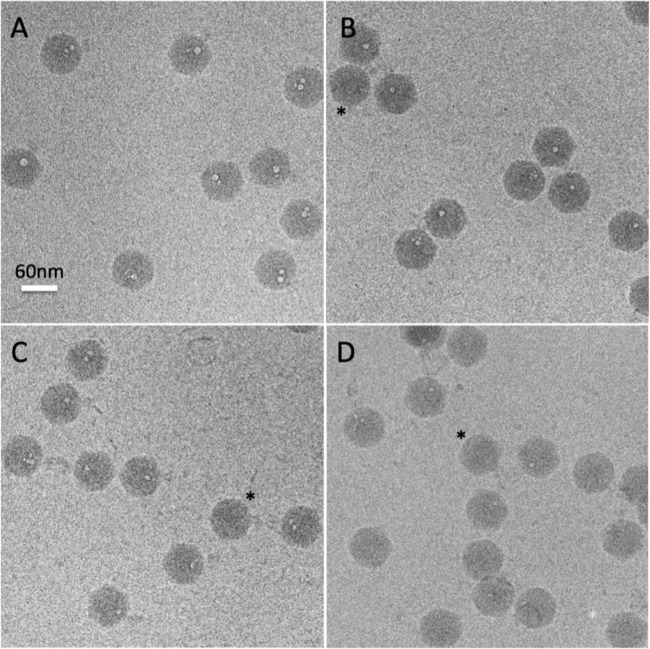
Cryo-EM of P22 virions with different E-protein contents. Particles with different E-protein contents exhibit different degrees of bubbling, after receiving the same amount of electron irradiation. To illustrate, the 8th exposure from each dose series is shown. (A) Wild type; (B) Δ20 mutant; (C) Δ16 mutant; (D) Δ7 mutant. Asterisks denote some virions that have not yet bubbled.

**FIG 3:**
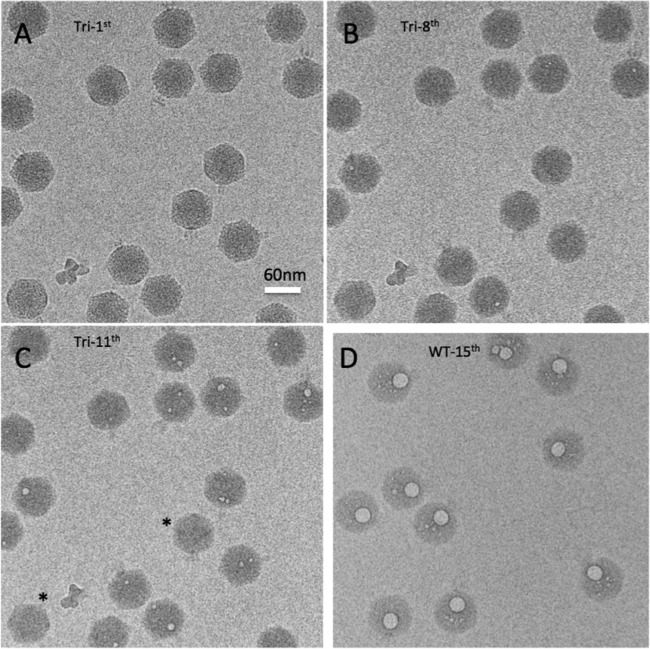
A cryo-EM dose series for P22 triple-deletion mutant virions. (A to C) A cryo-EM dose series for P22 TriΔ particles, lacking all three E-proteins. The 1st, 8th, and 11th exposures are shown. Note that the tails that are clearly defined in the first exposure, protruding laterally from the capsid, become progressively less well defined in exposures 8 and 11. Note also the different degrees of bubbling from virion to virion in exposure no. 11. In particles with more than one bubble, the bubbles are often quite far apart, indicating that secondary bubbles develop at approximately the same time as primary bubbles with this specimen. (D) Fifteenth exposure of wild-type virions, which uniformly contain one large, spherical, coalescent bubble per virion plus a few diminutive secondary bubbles that are often quite far from the primary bubble.

Wild-type P22 virions and each of the mutants were prepared for cryo-EM, and dose-series images were recorded (see Text S1 in the supplemental material). Each 1-s exposure corresponded to delivery of ~16 electrons/Å^2^, and there were 10-s pauses between exposures. A dose series consisted of up to 15 exposures. The morphologies (sizes and shapes) of all of the mutant particles were indistinguishable from the wild-type (Fig. 2 and 3).

Each kind of virion eventually exhibited bubbling but with different thresholds of the irradiation required (Fig. 2 and 3). Wild-type P22 bubbled earliest, with bubbles first appearing in the 5th exposure. The triple mutant was slowest, with bubbles appearing only in the 10th exposure. The bubbling thresholds of the three single mutants were intermediate between these extremes. Δ7 was the slowest single mutant, and Δ16 and Δ20 were approximately the same: slower than the wild type but faster than Δ7. These observations make it clear that the E-proteins are primarily responsible for the observed bubbling. However, the fact that bubbling eventually occurs in the triple mutant (Fig. 3) points to the presence of some other bubbling-competent material (protein). To illustrate this behavior, three exposures from a dose series of the triple mutant are presented in Fig. 3A to C. In the first (low-dose) exposure, the virions are sharply defined and tails are clearly visible, protruding laterally from the capsid. In the 8th exposure, the particles have become blurred, tails are barely visible, and a few virions show incipient bubbling. In the 11th exposure, bubbling is well established in most virions but a few still show no sign of bubbling. Figure S1 in the supplemental material shows fields of wild-type virions and the four mutants in which bubbling has advanced to a similar degree in each case, as the result of different amounts of irradiation (i.e., exposure number).

**FIG 4:**
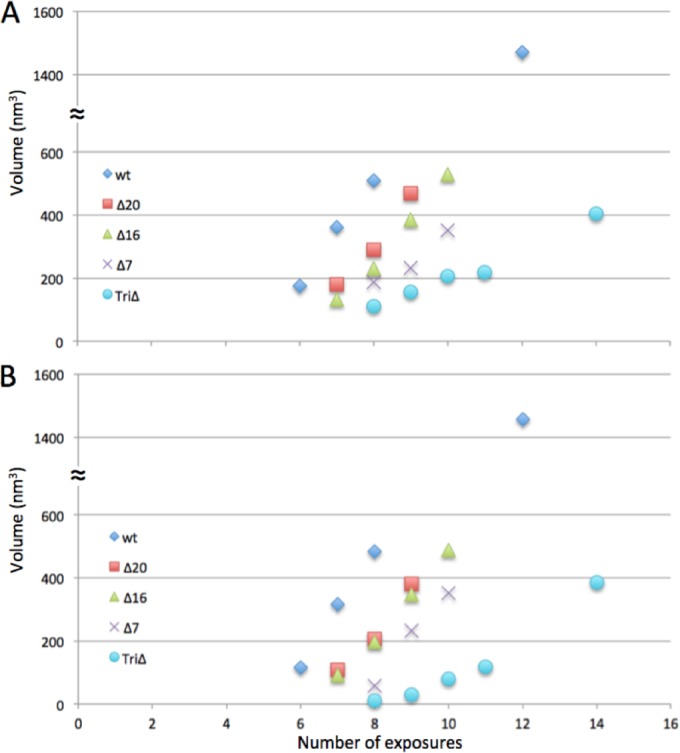
Bubble volume increases with increasing electron dose. (A) The data are plotted only for particles that have started bubbling. (B) The data are plotted for all particles. The *x* axes are labeled in exposure units (see Materials and Methods).

Bubbling was quantitated in terms of average total bubble volume per virion and plotted as a function of exposure number. This was done in two ways: counting only bubbling virions (Fig. 4A) and counting all virions (Fig. 4B). After a bubbling threshold is reached, the total bubble volume increases in an approximately linear fashion and at similar rates for all virions except the triple mutant, whose bubbles tend to be smaller and to grow less rapidly.

### Location of bubbles within the virion.

Referring to the tail as a fiducial marker for the portal vertex, we found that the bubbles are in each case internally situated at locations along the portal axis (Fig. 2 and 3). Eventually, secondary bubbles begin to appear at other internal sites (Fig. 3C and D), but they start later and remain much smaller than the primary bubbles, which continue to grow. Moreover, unlike the closely spaced members of a cluster of primary bubbles (which eventually merge [Fig. 3D]), secondary bubbles are often located far from the primary cluster (Fig. 3C and D). Over the dose range covered, no bubbling was seen in the capsid shell or the tail. We attribute this restriction of bubbling to internal locations to the effect of densely packed DNA retarding the outward diffusion of radiolytic products—in particular, hydrogen gas (40, 41); in this way, a critical concentration for bubble nucleation is reached earlier than with proteins that are not so confined (36). Thus, bubbling occurs more readily the deeper the proteins are embedded in DNA. The following analysis focuses on the primary bubbles.

Typically, in a P22 virion, a cluster of 1 to 3 small bubbles nucleates, and in subsequent exposures these bubbles grow and eventually coalesce (Fig. 3D). The clusters are located close to the portal axis. This trend is qualitatively similar to what occurs with bacteriophage T7 (36), although cryo-EM reconstructions of P22 have suggested (4) that it houses no close counterpart to the T7 core (11).

The degree of bubbling in a field varies somewhat from virion to virion (see Fig. 2D and 3A to C). For example, in exposure 6 of this dose series on wild-type virions, ~9% of the particles already exhibit substantial bubbles, up to 9 nm (average, ~7 nm) in diameter, while others have no discernible bubbles (data not shown). Some examples of bubbling-reluctant virions are marked with asterisks in Fig. 2C and D and 3C. Neighboring virions that exhibit bubbling have been subjected to the same irradiation and are embedded in an ice layer of essentially uniform thickness, both factors that could affect bubbling. (Bubbling occurs sooner for particles embedded in thinner ice [N. Cheng, unpublished results].) The observed variability may reflect the stochastic nature of bubble nucleation; however, an alternative possibility is that virions of a given genotype vary in their contents of E-proteins, with virions that have more E-protein bubbling earlier.

### The shape and location of the gas cloud depend on which E-proteins are present.

**FIG 5:**
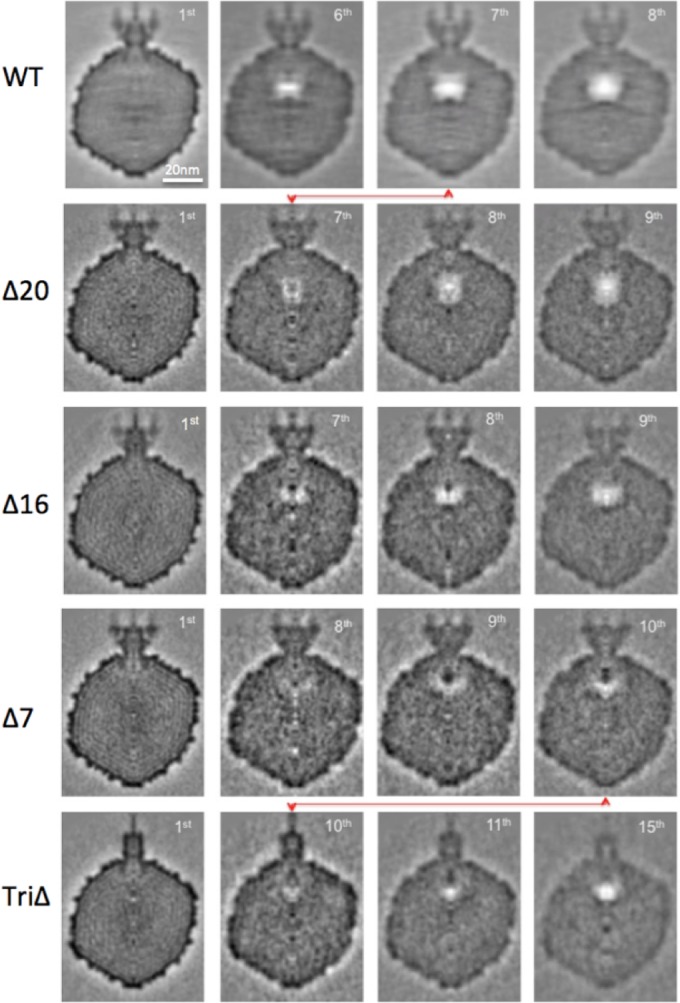
Development of gas clouds in wild-type P22 and mutant virions. Sagittal central sections from cryo-EM reconstructions are shown in gray scale for selected exposures of five dose series. The P22 genotype is indicated at the left of each row, and exposures increase from left to right. The exposure number used for each reconstruction is shown at the top right of each frame. In the triple mutant preparation that was analyzed, most of the virions lacked tail spikes; the reason for this lack is probably that the *galK* insertion (see Materials and Methods) causes poor tail spike expression, but the virions were otherwise normal. The red linkers associate sections from the same exposure in different mutants. Genotype labels are given at left.

In order to localize the bubbling more precisely and to chart its development, we calculated “asymmetric” reconstructions for each mutant at various stages of bubbling (see Text S1 in the supplemental material). In such a reconstruction, the particles are not subjected the 60-fold averaging that comes from imposing icosahedral symmetry. In all, 30 density maps were reconstructed. Central sections from selected reconstructions are presented in Fig. 5. Because of the limited amount of data per reconstruction (200 to 400 virions), the resolutions are relatively low (see Text S1) and further constrained by radiation damage and the fact that bubbles do not develop in an identical fashion in each virion (described above). Nevertheless, the reconstructions are informative regarding the distribution of E-proteins inside the virions.

When data are recorded at electron doses far enough beyond a bubbling threshold, the reconstruction contains a “gas cloud” (Fig. 5). This cloud represents the region that is occupied by gas in most (or all) of the virions included in the reconstruction. Typically, with P22, a gas cloud first appears in the reconstruction of images from the next exposure after the one in which bubbles are first observed in individual particles. Beyond this point, the gas cloud continues to grow, accompanied by progressive blurring of the virion structure outside the gas cloud (45) (Fig. 5).

**FIG 6:**
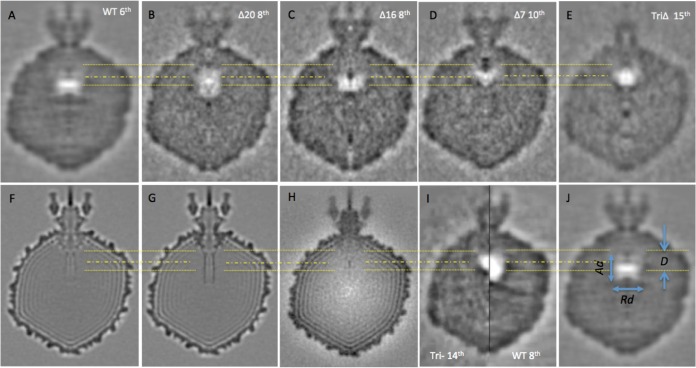
Central sections through reconstructions of P22 virions. Panels A through E are from bubblegram reconstructions in the present study. Panels F and H are from two published high-resolution low-dose reconstructions (48) (EMDB-1220) and (59) (EMDB-1222), respectively. Panel G has a model of the gp1 crystal structure (PDB code 3LJ5) band limited to a 15-Å resolution implanted in the relevant portion of panel F. Panel I illustrates with half-plane sections the axial offset between the gas clouds from the 8th-exposure wild type and the 14th-exposure TriΔ. Panel J defines the gas cloud dimensions: *D*, Ad, and Rd. The yellow transverse lines mark the axial positions of certain axial reference points to allow comparisons to be made. The upper yellow dotted line marks the position of the portal crown. The yellow dotted-and-dashed line marks the centroid of the TriΔ gas cloud. The bottom dotted yellow line marks the centroid of the wild-type gas cloud (see panel A).

The development of gas clouds in the five strains studied here is illustrated in Fig. 5. All of the gas clouds lie along the portal axis. The portal crown affords a reference point for specifying the positions of gas clouds out along the portal axis. (We refer to inner surface of the gp1 dodecamer, minus the barrel of C-terminal domains, as the “crown” [Fig. 6F].) The spacing from the center of a gas cloud to the crown is its displacement (*D*). Its extent along the portal axis is described by axial diameter (Ad) and, perpendicular to the portal axis, by radial diameter (Rd) (Fig. 6J). Some values of these parameters measured on selected reconstructions are given in Table 2. To facilitate comparisons, the upper dotted yellow line running through all panels of Fig. 6 represents the axial position of the portal crown (Fig. 6F); the dotted and dashed yellow line 5 nm below it marks the displacement of the TriΔ mutant, and the bottom dotted yellow line at *D* = *9* nm marks the displacement for wild-type virions.

**TABLE 2:** Gas cloud dimensions of selected P22 bubblegram reconstructions

Phage and exposure^a^	Value for gas cloud (nm)^b^		
	*D*	Ad	Rd
WT			
6th	9.0	7.0	10.6
7th	9.0	10.0	12.4
8th	9.0	12.4	14.2
TriΔ, 11th	4.5	4.7	7.2
Δ20, 7th	9.0	12.8	8.0
Δ16, 10th	9.0	9.5	12.0
Δ7, 10th	9.0	8.8	8.0

a “WT 6th” means the reconstruction of the wild-type virion from the 6th exposure.

b D is the displacement of the centroid of the gas cloud relative to the portal crown. Ad and Rd are, respectively, the extent (diameter) of the gas cloud in the axial and radial dimensions. See Fig. 6J.

In this dose series on wild-type virions, a gas cloud is first detected in the reconstruction from exposure 6. It has a displacement, *D*, of 9 nm and is slightly oblate (i.e., wider than it is long [Rd = 10.6 nm and Ad = 7.6 nm]). Two exposures later, *D* is unchanged, but the gas cloud is rounder and has expanded so that Rd =14.2 nm and Ad =12.4 nm. The gas clouds appearing in the Δ20 (Fig. 6B) and Δ16 (Fig. 6C) reconstructions have the same *D* value as the wild type. The Δ20 gas cloud is slightly prolate (i.e., longer than it is wide), while that of Δ16 is slightly oblate (i.e., wider than it is long).

The TriΔ triple mutant is last to develop a gas cloud. With 10th-exposure data (Fig. 5, bottom row), there is a small incipient cloud, and the first fully developed cloud comes with exposure 11. Its displacement, *D*, is 5.2 nm: i.e., its center is ~4 nm closer to the portal crown than the wild-type gas cloud (cf. Fig. 6A, E, and I). Its shape is also slightly prolate. Of note, the Δ7 cloud has the same axial displacement (*D* value) as the triple mutant (Fig. 6D and E, respectively).

## DISCUSSION

### The E-proteins are internal components of the P22 virion.

As the gas clouds are buried well below the surface of the virion (Fig. 5), the E-proteins must be located internally. This conclusion is supported by other, less direct, lines of evidence: eliminating them by deleting their genes allows increased amounts of DNA to be packaged (J. C. Leavitt, unpublished results), E-proteins are not degraded when virions are exposed to proteases (S. R. Casjens, unpublished results), and this property explains the observation (46) that acridine-mediated E-protein damage requires the presence of DNA in the virions.

Of the five genotypic variants analyzed, the wild-type virion is the first to bubble and the triple-deletion mutant is the last. It follows that E-proteins are the predominant contributors to bubbling in wild-type phages. Because all of the single mutants bubble earlier than the triple-deletion mutant (Fig. 4), we infer that all three E-proteins contribute to the bubbling. In the absence of all E-proteins, there is still bubbling-competent material inside the capsid (Fig. 3A to C), which we attribute to the barrel domain of the portal protein (see below).

### E-proteins make contact with each other and with the portal barrel domain.

The similar locations of the gas clouds of the various mutants (Fig. 5 and 6) imply that the three E-proteins are close together and may interact. On similar grounds, they are likely also to interact with the portal barrel domains. Interactions among E-proteins are further suggested by the SDS-PAGE data, which indicate that a virion’s complement of one E-protein is affected by the absence of another E-protein, particularly so in the case of gp7, in whose absence gp20 is greatly reduced (Fig. 1; Table 1). It is noteworthy that in wild-type virions, gp7 and gp16 have approximately the same copy number as the portal protein gp1. This would be readily explained if these proteins were to have binding sites on the portal.

### E-protein contents correlate with propensity to bubble.

We estimated the E-protein copy numbers by SDS-PAGE (Table 1) and the bubbling thresholds in terms of the exposure number at which bubbles were observable in 50% of virions (Table 3). Estimates of bubbling thresholds can vary by 1 exposure unit between ostensibly identical dose series (N. Cheng, unpublished) (Table 3). The reasons for this are not entirely clear, but differences in ice thickness and limited accuracy of dose calibration appear to be factors. A bubbling threshold also reflects the amount of E-proteins present; however, the relationship is not straightforward as some proteins are more bubbling-prone than others (38). Such appears to be the case for gp16 (Tables 1 and 3).

**TABLE 3:** Bubbling thresholds for wild-type and mutant P22 virions

Threshold expt no.	Threshold exposure no.^a^				
	WT	Δ20	Δ16	Δ7	TriΔ
Expt 1	5.6	6.7	6.5	8.8	10.7
Expt 2^b^		5.6	5.7		

a The units given are exposure numbers at which 50% of the virions in that member of a dose series are bubbling. Fractional exposure numbers are calculated as follows: for instance, if 30% are bubbling at exposure 5 and 60% are bubbling at exposure 6, we call that 5.7 (5 × 0.33 + 6 × 0.67).

b In experiment 2, analyses of Δ20 and Δ16 were repeated. Again, the results were equal, although there was a difference of approximately 1 exposure from experiment 1 despite our best efforts to standardize the procedure.

Although the E-proteins add up to about 2.5 MDa (Table 1), the cryo-EM reconstructions reported to date have not detected substructures large enough to account for that amount of protein. This suggests that the E-protein locations vary sufficiently for their densities to be smeared out in reconstructions. For instance, the proteins may bind to specific locations on the interior surface of the capsid, but with most of their mass (folded or unfolded) offset by flexible linkers. The narrow bore of the portal channel (~3.5 nm [17] or less if side chains lining the channel are taken into consideration) suggests that E-proteins are at least partly disordered when they pass down through the portal channel en route out of the virion. The issue of transient unfolding has been discussed for ejected proteins of bacteriophage T4 (5).

Next we consider the interpretability of gas clouds and properties of the portal barrel and then go on to discuss the implications of the bubblegram data for the locations of the E-proteins and their interactions with portal barrel domains: inescapably, these models involve at least partial disordering of the barrel.

### Inferences from gas clouds.

The coordinates of the centroid of a gas cloud in the capsid frame of reference can be determined with precision. These coordinates remain robust through subsequent exposures in which the cloud continues to grow, approximately isotropically, eventually becoming spherical (Table 2; Fig. 3D). We assume that the gas cloud centroid coincides with that of the local pool of bubbling-competent protein. It is not possible to relate the size of a gas cloud directly to that of the proteinaceous structure(s) producing it, as the cloud continues to grow while the size of the latter is fixed. Although gas cloud shapes do not yield detailed structural information, departures from sphericity offer clues as to the distribution of the bubbling protein (see the oblate and prolate gas clouds described above).

### The E-proteins are distributed around the portal axis, centered on a point ~9 nm in from the portal crown.

**FIG 7:**
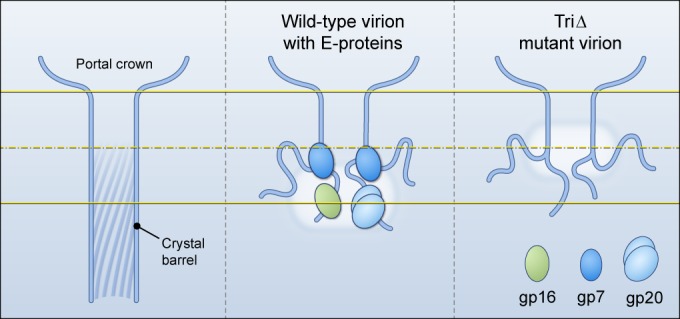
Schematic models of proposed arrangements of the portal barrel domains. The full-length 12-stranded coiled-coil barrel of the crystal structure (PDB code 3LJ5) is shown in the left panel, compared with its proposed dispositions in virions with and without E-proteins (center and right panels). For clarity, only one or two copies of the disordered barrel domain and E-protein subunits are shown. The fuzzy white clouds represent the positions of gas clouds. Transverse yellow lines mark axial reference points as in Fig. 6.

The bubblegram reconstructions (Fig. 5 and 6) show clearly and consistently that the presence of E-proteins in the wild-type and mutant virions correlates with gas clouds centered on the portal axis. Their centers are positioned ~9 nm in toward the virion center from the portal crown in the cases of the wild type, Δ20, and Δ16 and ~4.5 nm in from the portal crown in the cases of Δ7 and TriΔ. If the barrel in the TriΔ virion were to be complete (i.e., as in the crystal structure), the associated gas cloud should be near its tip, as this is the region most deeply embedded in DNA so that the efflux of radiation-induced H gas is most effectively impeded, thereby promoting the buildup of gas to a critical concentration for bubbling (36). However, the tip is 15 nm from the crown, whereas the center of the TriΔ gas cloud is only 4.5 nm from the crown. To place barrel domain density in the gasifying region, we envisage that their distal portions are unfolded, leaving a shorter barrel. Similarly, the wild-type gas cloud is further from the crown than the TriΔ gas cloud, suggesting that gp16 and gp20 are located beyond the end of such a shorter barrel. The proposed arrangement is shown in Fig. 7.

It has been shown that the E-proteins exit from the capsid before the DNA (26). Assuming that they exit via the portal barrel, it is plausible that the E-proteins should initially reside at or close to the distal end of the barrel. Several observations suggest that the E-proteins interact with the barrel domain (described above). gp7 appears to be the primary binder. gp20 needs gp7 in order to be incorporated efficiently—probably as dimers or trimers—into the assembling virion. Incorporation of gp16 is assisted by gp7 but is less dependent on it than is gp20. The oblate shape of the wild-type cloud and the prolate shape of the Δ20 gas cloud suggest that the gp20 subunits are mostly further out from the portal axis (Fig. 7).

Two other observations support models of a shorter barrel connecting to a disordered distal region. (i) The last 50 residues (676 to 725, deleted in the dif1 mutant) of gp1 are dispensable (47). In contrast, all three E-proteins are essential, suggesting that their putative binding sites on gp1 are N-terminal to residue 676. The dif1 mutant with its truncated gp1 still contains the E-proteins (S. R. Casjens, unpublished). If the C-terminal part of gp1, deleted in dif1, is disordered in the wild-type virion (as we propose), that would shorten the barrel by about 7 nm. (ii) According to a predictive algorithm, the last 70 residues of gp1 have little propensity for coiled-coil conformation, unlike the preceding 40 residues that have a strong propensity (8). This observation is also consistent with the distal region being disordered.

### Conformation and interactions of the portal barrel domain *in situ*.

The P22 virion has been visualized in several asymmetric cryo-EM reconstructions, but different interpretations have been made. Sagittal slices through two reconstructions of the wild-type virion are shown in Fig. 6F and H. In their reconstruction, Lander et al. (48) found several irregular rings stacked along the portal axis: when 12-fold symmetry was applied, it gave a faint tubular density, about 9 nm long, extending from the portal crown (Fig. 6F). However, no such density was seen in another reconstruction of virions at about the same resolution (Fig. 6H), suggesting an even greater degree of disorder than we have invoked. Lander et al. originally interpreted their tubular density as E-protein, but after the portal protein crystal structure was determined (18), Tang et al. (8) reinterpreted this density as the portal barrel.

To assess the portal barrel in the context of the virion, we replaced the relevant region of the reconstruction computationally with the crystal structure of gp1 limited to a 15-Å resolution (Fig. 6G). The two structures match reasonably well (cf. Fig. 6F and G), but the virion cylinder is markedly shorter (9 nm) than the crystal barrel (15 nm in the part of uniform diameter). Due to the low contrast and an adverse signal-to-noise ratio, it is difficult to measure the tube length precisely from Fig. 6F, but it is consistent with the shortened barrel discussed above, perhaps decorated with some E-protein around the rim.

### E-proteins enter the capsid at the stage of procapsid assembly.

An asymmetric reconstruction of the P22 procapsid (15) showed only a short cylindrical segment, ~4 nm long, at the base of the barrel. The rest was presumably disordered. Similarly, no barrel-like density was detected in reconstructions of the isolated tail machine (49), indicating that the C-terminal region of gp1 is flexible and at least partly unfolded under those conditions.

Scaffolding protein is required for E-proteins to be incorporated into the procapsid, but portal is not (21, 50–52). In light of these observations, we conjecture that E-proteins bind to portal inside the assembling procapsid after some scaffolding protein is in place, before the particle is completed.

## MATERIALS AND METHODS

### Bacteriophages.

Isogenic E-protein gene deletion mutant P22 phages were constructed as prophages in *Salmonella enterica* LT2 using *galK* recombineering as described in references 53, 54, and 55. Details of construction and full genotypes of the phages are given in Text S1 in the supplemental material. Briefly, prophages missing ejection protein gene *7*, *16*, or *20* or all three of these genes (referred to as simply Δ7, Δ16, Δ20, and TriΔ, respectively) were made in which each gene was neatly deleted, except that in the gene *7* and *20* deletions, the 3′-terminal 60 bp of the coding region was not removed. Thus, none of the deletions removed the ribosome binding signals of the nearby downstream gene, and the unaltered genes should be expressed normally. Phage particles were produced by induction of prophages to lytic growth with mitomycin C or by infection for wild-type particles, and highly purified DNA-containing particles were prepared through two successive CsCl step gradient centrifugations (56) (see Text S1 for details).

### Cryo-electron microscopy and image reconstruction.

Specimens were prepared for cryo-EM, and dose series of micrographs were collected on a CM200-FEG electron microscope (FEI), essentially as described previously (36). The first exposure was recorded on film, further exposures until the onset of bubbling were recorded on a charge-coupled device (CCD) camera, and then five more exposures were recorded on film. Images recorded on film were digitized and binned 2-fold, giving 3.34 Å/pixel. EMAN1 (57) and EMAN2 (58) were used for image processing. Contrast transfer function zeroes were determined from 1st-exposure images and used to perform a phase-flipping correction on all images in that series. To align the images in a given series, the centers of three particles were used as reference points to calculate the translation and rotation parameters needed to bring subsequent exposures into alignment. In calculating reconstructions, capsid orientations were determined for the 1st-exposure images and applied throughout the dose series. Initially, we assumed icosahedral symmetry, and this yielded 12 possibilities for the location of a tail; the portal vertex was then identified using a tailed model. For ambiguous particles, additional calculations were performed (see Text S1 in the supplemental material). Asymmetric reconstructions were calculated according to reference 11; finally, C5 symmetry was enforced. The numbers of particles in each data set and the resolutions obtained, as assessed by Fourier shell correlation, are given in the supplemental material.

### Accession number(s).

Representative density maps from the 1st, 7th, and 8th exposures of the Δ20 dose series have been deposited in the EMDB database as EMD-8258, EMD-8259, and EMD-8260, respectively. The other density maps are available on request.

## SUPPLEMENTAL MATERIAL

Text S1  The genetic, molecular biological, and biochemical methods employed in this study are described, as are the procedures for cryo-electron microscopy and three-dimensional image reconstruction. Download Text S1, DOCX file, 0.2 MB

Figure S1  The cumulative electron dose required to generate bubbles of a given size in P22 virions varies according to genotype. Shown are cryo-electron micrographs of (A) the wild type at 6th exposure, (B) Δ20 at 7th exposure, (C) **Δ**16 at 7th exposure, (D) Δ7 at 9th exposure, and (E) TriΔ at 11th exposure. In each case, the bubbling varies among individual virions. Virions that have yet to start bubbling are marked with asterisks. Download Figure S1, PDF file, 1.2 MB
