# Supplementary material for: Localization of the Houdinisome (Ejection Proteins) inside the Bacteriophage P22 Virion by Bubblegram Imaging
Source: mBio. 2016 Aug 9;7(4):e01152-16. doi: 10.1128/mBio.01152-16 (PMC4992974; doi:10.1128/mBio.01152-16)
Supplement: Text S1 — The genetic, molecular biological, and biochemical methods employed in this study are described, as are the procedures for cryo-electron microscopy and three-dimensional image reconstruction. Download [file mbo004162935s1.docx]

**Wu et al : Localization of P22 ejection proteins.**

**Supplementary material, Methods- S1**

**Materials & Methods.**

**Bacteria and phages.** Isogenic E-protein mutant P22 phages were constructed for this study as follows: The *galK* recombineering methods used are described in references ([53-55](#_ENREF_53)). The pKD46 plasmid (61) was present to promote recombinational replacement, but was removed by growth at 41°C before prophage induction. *Salmonella enterica* serovar Typhimurium LT2 strain UB-2235 is *galK*^–^, lacks prophages Fels-1, Fels-2, Gifsy-1 and Gifsy-2, and carries a P22 UC-937 prophage (genotype P22 *c1*-7, *13^–^am*H101, *sieA*Δ-1, orf25::CamR-EG1; its construction will be described later (S.R.C. and E.B.G – to be published). The *c1*-7 mutation greatly lowers the frequency of lysogen formation so that the phage grows essentially lytically in liquid culture, *13^–^am*H101 blocks normal lysis and gives higher phage yield, *sieA*Δ-1 removes Mnt repressor binding sites that lower tailspike protein production, and orf25::CamR-EG1 is a chloramphenicol resistance cassette that replaces non-essential DNA between genes *15* and *3.* Since none of the mutations in UC-0937 affect virion assembly, it is referred to as "wild type" (WT) in this study. The *galK* cassette was amplified from plasmid pGalK (62) with three sets of primers that allow recombinational insertion of the amplified DNA into the P22 UC-937 prophage of UB-2235 to generate strains UB-2276, UB-2274 and UB-2272 in which the *galK* cassette replaces gene *7, 16* or *20*, respectively. Synthetic oligonucleotides were designed so that their recombinational replacement of these *galK* insertions generated prophages lacking essentially all of the E-protein genes. These deletions each neatly remove the entire coding region of each gene except in the gene *7* and *20* deletions the 3'-terminal 60 bp of the coding region was not removed; this should allow normal translational initiation of the downstream gene. Thus, the P22 prophages of the resulting strains, UB-2289, UB-2288 and UB-2285, carry *7^–^*∆7-1, *16^–^*∆16-1 and *20^–^*∆20-1 alleles in addition to the other mutations described above; these are referred to in the text as simply ∆7, ∆16 and ∆20, respectively. A mutant bacterial strain UB-2278 that harbors a prophage (called "tri∆") that lacks all three E-protein genes was made by inserting the *galK* cassette between P22 bps 6368 and 13254 so that it replaces all of genes *7* and *20* and all of gene *16* except its 60 3'-bp). The structure of each of the phage genomes mentioned above was confirmed by DNA sequencing of the modified regions.

Phage particles were prepared as follows: prophages were induced from exponentially growing cells at 2x10^8^ cells/ml, 37°C, in broth cultures of the above lysogens by addition of 1.5 μg/ml carbodox, with continued shaking for 5 hr. These cultures were lysed by shaking with chloroform and, after a cell debris-removal low speed centrifugation, virions were purified by two successive CsCl step gradient centrifugations (56) and dialysis against 1 mM MgCl_2_, 10 mM TrisCl, pH 7.4 buffer. Genuinely wild-type phages (without the mutations in strain UC-937 above, but with the c1-7 mutation that prevents lysogeny) were also generated from an infection of *Salmonella* strain DB7136 with wild-type phage. The phages were pelleted by ultracentrifugation and purified by CsCl step gradient centrifugation followed by dialysis into buffer as described above.

**Cryo-electron microscopy**. Typically, a 3.5-µl drop of specimen was applied to an EM grid bearing a thin carbon film, then blotted to a thin film and vitrified, using a Leica EM GP cryo-station. Dose series of images were collected on a CM200-FEG transmission electron microscope (FEI), operated at 120 keV and 38,000x magnification, essentially as described ([36](#_ENREF_36)). For each sample, after the first exposure (which was recorded on film), further exposures on a CCD camera (model US1000, Gatan) were used to detect the onset of bubbling, at which point five more exposures were recorded on film. (This medium was used to capture more particles). The exposure time and dose were the same with film or CCD camera. Data were recorded as follows: Wild-type - 1st, 6th, 7th, 8th, 14th, 15th exposed on films (6 dose series); TriΔ - 1st, 8th, 9th, 10th, 11th, 14th, 15th (6 dose series); Δ16 - 1st, 7th, 8th, 9th, 10th, 11th (6 dose series); Δ7 - 1st, 8th, 9th, 10th, 11th, 12th (6 dose series); Δ 20 - 1st, 7th, 8th, 9th, 10th, 11th (6 dose series).

**Image reconstruction.** Micrographs were digitized on a Nikon Super Coolscan 9000 scanner with a 6.35 µm step size and binned 2-fold, giving a sampling rate of 3.34 Å/pixel. EMAN1 (57) and EMAN2 (58) were used for image processing. To align the images in a given series, the centers of three particles on each image were marked. These three points define a triangle whose circumcenter was used to define a reference point on each image, while the vector from the circumcenter to one of the three particles was employed to calculate the rotation matrix needed to bring the images into alignment. The resulting translation and rotation parameters were used to align the particles from subsequent exposures with those of the 1st exposure. All the usable particles in a given series were picked with *e2boxer.py* and *batchboxer*. The zeroes of the contrast transfer function (CTF) were determined from the 1st-exposure images and used to perform the same phase-flipping correction on all the images in that series. The particles’ centers (origins), initially estimated as described above, were refined using *cenalignInt* in EMAN1.

To calculate reconstructions, capsid orientations were first determined by projection-matching, focusing on the 1st-exposure images and applying these results throughout the dose series. Initially, we assumed icosahedral symmetry and this yielded twelve symmetry-related possibilities for the location of the tail on any given particle. In order to locate its portal vertex (and thus the portal axis), we computed a projection for each of the 12 candidate orientations, using a tailed model calculated from a subset of particles whose tails were clearly evident. By comparing the positions of the tail in these projections and in the corresponding image, the portal vertex could be clearly identified in most cases. For ambiguous particles whose tails were eclipsed (a minority), we relied on the nucleating bubbles, which were assumed to be adjacent to the portal vertex, to identify that vertex. We justify this provision as follows. In thin ice films, most P22 virions orient so at to present side-views, with the tail protruding to the side and clearly visible. This orientation keeps the whole particle hydrated. On these virions, nucleating bubbles are seen to be close to the portal axis and about 9 nm in from the portal crown. As this pattern was consistently observed, we concluded that, for virions that had no visible tail, the nucleating bubbles role could serve as fiducials. Asymmetric reconstructions were then calculated according to ([11](#_ENREF_11)), without enforcing any symmetry. The orientations found for particles in low-dose (first exposure) images were assigned to the corresponding particles in later exposures in the same dose-series. The data sets contained 463 particles for wild-type P22, 262 particles for ∆20, 379 particles for ∆16, 534 particles for ∆7, and 247 particles for tri∆. Finally, C5 symmetry was enforced for analysis and presentation.

As assessed by the Fourier Shell Correlation, the first exposure reconstructions had resolutions of 37 – 40 Å, decaying to 56 – 63 Å for the 8^th^ exposure, and ~ 70 Å for 12^th^ exposures and higher.

Deposition: The reconstructed density maps from the 1^st^, 7^th^ and 8^th^ exposures of the Δ20 dose series have been deposited in the EMDB as EMD-8258, EMD-8259, EMD-8260, respectively, considered as representative of this large series (n = 30) of relatively low-resolution reconstructions. The other density maps are available on request.
