# Supplementary material for: Localization of the Houdinisome (Ejection Proteins) inside the Bacteriophage P22 Virion by Bubblegram Imaging
Source: mBio. 2016 Aug 9;7(4):e01152-16. doi: 10.1128/mBio.01152-16 (PMC4992974; doi:10.1128/mBio.01152-16)
Supplement: Figure S1 — The cumulative electron dose required to generate bubbles of a given size in P22 virions varies according to genotype. Shown are cryo-electron micrographs of (A) the wild type at 6th exposure, (B) Δ20 at 7th exposure, (C) Δ16 at 7th exposure, (D) Δ7 at 9th exposure, and (E) TriΔ at 11th exposure. In each case, the bubbling varies among individual virions. Virions that have yet to start bubbling are marked with asterisks. Download [file mbo004162935sf1.pdf]

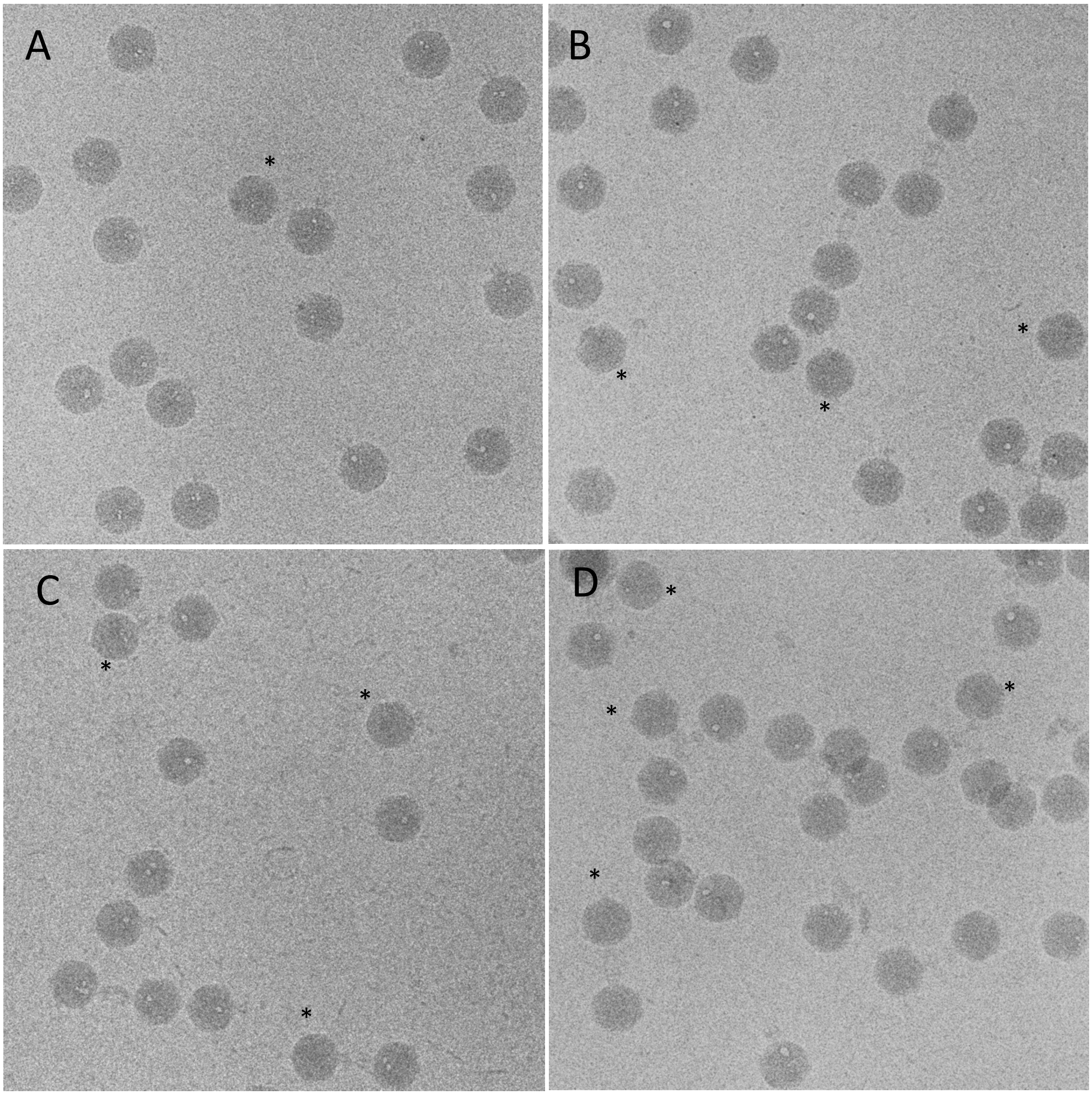

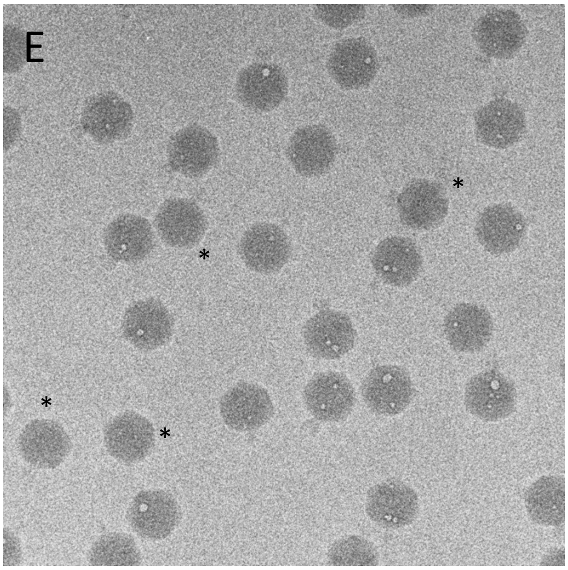

Supp. Fig S1

Different electron doses are required to generate similarly size bubbles on P22 virions of the following genotypes. (A) wild type at 6th exposure; (B) Δ20 at 7th exposure; (C) Δ16 at 7th exposure; (D) Δ7 at 9th exposure; (E) Triple mutant TriΔ at 11th exposure. In each case, the bubbling varies among individual virions. Some virions that have yet to reach their bubbling thresholds are marked with asterisks.
